# An Outbreak Investigation of Varicella Zoster among Male Military Personnel in a Military Training Centre

**DOI:** 10.31729/jnma.7440

**Published:** 2022-05-31

**Authors:** Parag Karki, Lee Budhathoki, Anita Poudel, Swojay Maharjan, Manoj Khadka, Lochana Shrestha, Leela Paudel

**Affiliations:** 1 Department of Internal Medicine, Shree Birendra Hospital, Chhauni, Kathmandu, Nepal; 2 Department of Community Medicine, Nepalese Army Institute of Health Sciences, Sanobharyang, Kathmandu, Nepal; 3 Nepalese Army Institute of Health Sciences, Sanobharyang, Kathmandu, Nepal

**Keywords:** *chickenpox*, *military*, *outbreak*

## Abstract

**Introduction:**

Chickenpox is an acute, highly infectious disease. Outbreaks can occur in adults in closed institutional settings like hostels and barracks. This study aims to report varicella zoster outbreak among male military personnel in a military training centre.

**Methods:**

An outbreak investigation of chickenpox in a military training school and tertiary hospital was conducted. Ethical approval was taken from the Institutional Review Committee (Reference number: 267). Data was collected from February 5, 2020 to April 10, 2020 by questionnaire and clinical examination. Case definitions were prepared as per the Centre for Disease Control and Prevention criteria. Data was entered and analysed in Microsoft Excel 2010.

**Results:**

Out of the 167 male personnel in the training centre, 90 (53.89%) were susceptible to varicella and 84 (50.29%) were close contacts. The secondary attack rate of varicella zoster was 18 (21.43%). The mean age of the patients was 22.33±1.72 years. The mean days of admission were 5±2.28 days. The most common clinical features were generalised rash in 18 (100%), fever in 15 (83.33%), and body ache in 12 (66.67%) cases.

**Conclusions:**

Natural history, clinical, and epidemiological features of varicella zoster in a closed institutional setting was studied with the secondary attack rate being less as compared to other studies.

## INTRODUCTION

Chickenpox (Varicella) is an acute, highly infectious disease caused by primary infection of the varicellazoster virus with an incubation period ranging from 10 to 21 days characterised by fever, malaise, and itchy maculopapular rash.^1,2^ Cases are common in winter and spring with outbreaks occurring every 2 to 5 years.^1^

Varicella outbreaks are frequently reported in countries like India and Nepal where varicella vaccination is not a part of the country's routine immunisation schedule.^3^ Outbreaks are common in young school-going children however they can occur in adults in closed institutional settings like hostels and military barracks.^3,4^ Varicella in military barracks is an important health issue that needs to be addressed since adults are the high-risk groups for more severe diseases and complications like pneumonia, encephalitis, cerebellar ataxia, and bacterial superinfections.^1^

This study aims to report the findings of varicella zoster outbreak in one of the military training centres of Bhaktapur and the army hospital of Kathmandu.

## METHODS

This was an outbreak investigation reporting all the cases during an outbreak of chickenpox in the military training centre, Shree Mahabir Ranger Regiment Training School, situated in Nagarkot, and those admitted to Shree Birendra Hospital (SBH), a tertiary care hospital of Nepal Army, from the same training school. The training school was conducting a Ranger Basic training which is a training of 5 to 6 months duration. Ethical approval was taken from the Institutional Review Committee (IRC) (Reference number: 267) of the Nepalese Army Institute of Health Sciences (NAIHS). The first case (index case) was reported to Shree Birendra Hospital on February 5, 2020 and investigation was started from the same date till April 10, 2020. Confirmation of the outbreak was done by comparing the incidence of varicella of this year with the previous three years' data during the same period for the training centre.

The centre was visited for clinico-epidemiological study and interviews with the cases. Listing of all people residing in the centre was done along with observation of the living area, dining area, and classrooms. History was taken by face-to-face interviews with the cases using the questionnaire. All the susceptible personnel were observed for new cases till 42 days from last reported case, which corresponds to twice the longest incubation period.^5^

The Secondary Attack Rate (SAR), defined as the probability that an infection occurs among susceptible people within a specific group that is close contacts, household, barracks or other closed population, calculated by using formula (Number of cases among contacts of primary cases/Total number of contacts)x 100%.^6,7^

Case definitions used during the outbreak investigation were as per Centres for Disease Control and Prevention (CDC) criteria which define the clinical case as the one with acute onset of diffuse maculopapular vesicular rash among personnel in the training centre during the study period without other apparent cause, a probable case as the one meeting the clinical case definition in the training centre but not laboratory-confirmed nor epidemiologically linked to another probable or confirmed case, and confirmed case being the case which met clinical case definition and epidemiologically linked to another probable or confirmed case in the training centre.^8^

Close contacts were defined as a person who had direct contact with the case, indoor or outdoor. It is also defined as being within a distance of less than one metre from a case and or had talked for more than three words with a case and/or had face-to-face contact with the case.^9^

Susceptibles were those persons with no known history of varicella or vaccination against varicella in the training centre. If a confirmed case occurred, the case was admitted in SBH under isolation and was managed with antipyretics and tablet acyclovir 800 mg along with symptomatic treatment. The data was entered and analysed in Microsoft Excel 2010.

## RESULTS

There were a total of 167 male personnel in the training centre. Seventy-seven (46.11%) personnel had a past history of varicella and no one was vaccinated against varicella so a total of 90 (53.89%) personnel were susceptible to getting chickenpox. The trainees were residing in four rooms, with less than one metre bed gap and poor ventilation.

One hundred and twenty-five (74.85%) personnel in the centre were trained together and taking classes in one classroom. Out of 90 susceptible, 68 (75.56%) were trainees and all were in close contact with cases. Out of the remaining 22 (24.44%) susceptible, comprising of trainers and other staff, 16 (17.78%) were in close contact with at least one case. Thus, out of total 167 in the center, there were 84 (50.30%) close contacts in this outbreak.

**Figure 1:**
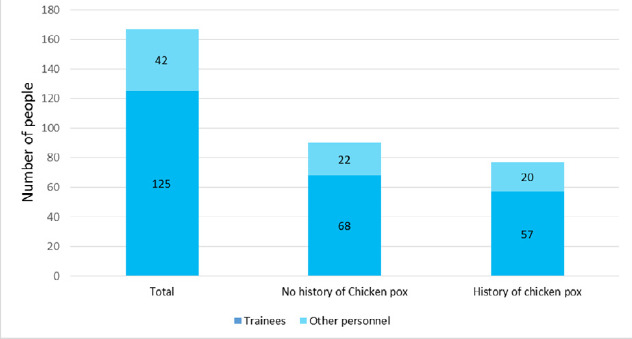
Bar graph showing total person in training centre with past infection status (n = 167).

The secondary attack rate of varicella zoster was 18 (21.43%). The breakdown of trainees and other personnel that included trainers, cooking, and cleaning staff and security personnel of the centre along with past infection status is shown below (Figure 1).

**Figure 2:**
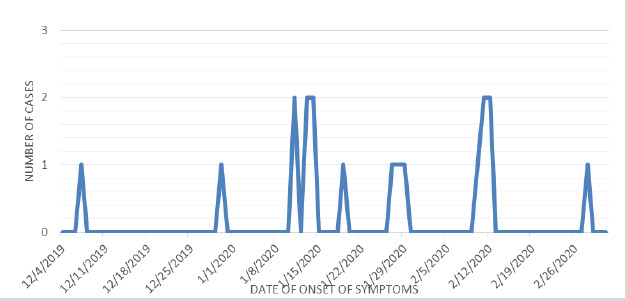
Epidemiological curve of the outbreak

On backward contact tracing, the primary case of varicella was found to occur retrospectively on December 7, 2019. The epidemiological curve for case distribution over time shows that the last case occurred on February 28, 2020, so susceptibles were observed till April 10, 2020, which corresponded to 42 days. All the cases were trainees with cases being distributed in all four rooms (Figure 2).

Among the cases, the most common first symptom seen was headache in 7 (38.89%) cases (Table 1).

**Table 1:** First symptom of chickenpox observed among cases (n = 18).

Symptoms	n (%)
Headache	7 (38.89)
Rash	6 (33.33)
Fever	3 (16.67)
Backache	1 (5.55)
Chills	1 (5.55)

The most common clinical feature observed was generalised rash in 18 (100%) cases, followed by fever 15 (83.33%) cases and body ache 12 (66.67%) cases. An average temperature of 37.80°C (100.05°F) was seen (Table 2).

**Table 2:** Clinical features of chickenpox among cases (n = 18).

Symptoms	n (%)
Fever	15 (83.33)
Rash	-
Localised	-
Generalised	18 (100)
Cough	7 (38.89)
Body ache	12 (66.67)
Sore throat	3 (16.67)
Headache	9 (50.00)
Malaise	10 (55.56)

The pattern of rash spread was centripetal in 12 (66.67%) and centrifugal in 6 (33.33%) cases. Mean duration of rash was 7.67±3.05 days. The rash was noted firstly in the head, neck, and face in 8 (44.44%) cases. No complications associated with chickenpox were reported in the cases in this outbreak (Table 3).

**Table 3:** Appearance of first rash in the body (n = 18).

Part of the body	n (%)
Head and neck	8 (44.44)
Torso	7 (38.89)
Extremities	3 (16.67)

## DISCUSSION

The outbreak occurred in trainees of a training centre in Ranger Regiment who are adult army personnel undergoing harsh training and live together in barrack structure thus making them susceptible to the spread of varicella which is a highly communicable disease. Sharing of the same classroom, dining hall, sports ground, training requirements requiring physical contact between trainees, before implementing the preventive measures, and prolonged harsh training with training period of Ranger basic training lasting for five to six months to meet standards of ranger regiment caused the further spread of the disease. Additionally, this outbreak occurred parallel to COVID-19 pandemic so leaves were suspended in the training of Nepal Army at that phase. Similar outbreaks have been reported in India and Singapore.^4,9^ The primary case in this outbreak was a trainee who had no history of contact with a case of varicella-zoster however he had a history of sharing clothing and close contact with another trainee with Herpes zoster. Herpes zoster causing varicella has been reported in the literature so the index case could have got an infection from the case of herpes zoster.^10^

The World Health Organisation (WHO) reports a secondary attack rate of chickenpox as 61-100%.^1^ Other studies reported SAR as 84%.^9^ The secondary attack rate in our study was less compared to these studies which could be due to the interventions done in this outbreak. As soon as the index case was reported, classes and closed activities were suspended. Ventilation was improved and overcrowding was reduced by increasing the number of rooms for accommodation as well as putting the bed of one person with a past history of varicella between two susceptible persons. Daily screening for symptoms and chicken pox drill was done.

The outbreak occurred in the winter season which is in line with other studies which report lower temperature increasing susceptibility to infection.^9,11,12^ Our study reports the most common clinical features as rash, fever, and malaise which is similar to that reported by other two studies done in similar setting.^3,9^ All our cases had a generalised rash which is similar to that reported by other studies.^3,13^ Fever was reported in a majority of cases i.e 96.4% cases, 83% casses and 75% cases in three different studies done in similar setting which is similar to that reported in ours.^3,9,13^

The study's strength is that it looked at an ongoing epidemic in a restricted institutional setting where participants were not able to leave owing to training obligations. The lack of lab confirmation of the cases is a limitation of this study; nonetheless, experts from Shree Birendra Hospital's Dermatology Department confirmed the diagnosis of varicella. Serosurveillance among trainees was also not checked to determine the immunity of close contacts and was exclusively reliant on history. Lastly, because the epidemic was studied in a confined military environment, the findings can only be generalised to similar settings.

## CONCLUSIONS

The secondary attack rate observed in this outbreak was lower than that reported by other literature which could be due to preventive measures being applied and strict implementation of them in a military training setup. This study has helped to study the natural history of varicella in a closed institutional setting and observe the clinical and epidemiological features in an ongoing outbreak.
